# Baseline investigation on soil solidification through biocementation using airborne bacteria

**DOI:** 10.3389/fbioe.2023.1216171

**Published:** 2023-06-14

**Authors:** Meiqi Chen, Sivakumar Gowthaman, Kazunori Nakashima, Chikara Takano, Satoru Kawasaki

**Affiliations:** ^1^ Laboratory of Biotechnology for Resources Engineering, Graduate School of Engineering, Hokkaido University, Sapporo, Japan; ^2^ Department of Engineering Technology, Faculty of Technology, University of Jaffna, Kilinochchi, Sri Lanka; ^3^ Laboratory of Biotechnology for Resources Engineering, Faculty of Engineering, Hokkaido University, Sapporo, Japan

**Keywords:** airborne bacteria, microbial induced carbonate precipitation, urease activity, bacterial identification, unconfined compressive strength

## Abstract

Microbial induced carbonate precipitation (MICP) through the ureolysis metabolic pathway is one of the most studied topics in biocementation due to its high efficiency. Although excellent outcomes have proved the potential of this technique, microorganisms face some obstacles when considering complicated situations in the real field, such as bacterial adaptability and survivability issues. This study made the first attempt to seek solutions to this issue from the air, exploring ureolytic airborne bacteria with resilient features to find a solution to survivability issues. Samples were collected using an air sampler in Sapporo, Hokkaido, a cold region where sampling sites were mostly covered with dense vegetation. After two rounds of screening, 12 out of 57 urease-positive isolates were identified through 16S rRNA gene analysis. Four potentially selected strains were then evaluated in terms of growth pattern and activity changes within a range of temperatures (15°C–35°C). The results from sand solidification tests using two *Lederbergia* strains with the best performance among the isolates showed an improvement in unconfined compressive strength up to 4–8 MPa after treatment, indicating a high MICP efficiency. Overall, this baseline study demonstrated that the air could be an ideal isolation source for ureolytic bacteria and laid a new pathway for MICP applications. More investigations on the performance of airborne bacteria under changeable environments may be required to further examine their survivability and adaptability.

## 1 Introduction

In the field of geoengineering, biocementation is a branch that applies microbial activity into soils to improve the mechanical properties, causing a cementation effect by microbial induced carbonate precipitation (MICP) through a wide range of metabolic pathways such as ureolysis, photosynthesis, ammonification, denitrification, sulfate reduction, and methane oxidation (Castro-Alonso et al., 2019; Hoffmann et al., 2021). As a promising technology, considerable achievements have been attained in the fields of environmental remediation, construction restoration, and geoengineering. In the area of environmental engineering, this technology has been widely studied as a method to contain hazardous wastes in polluted materials and prevent them from further leaching into clean waterbody (Kumar et al., 2023; Wang et al., 2023; Xue et al., 2023). For construction restoration, the conservation work should not alter the aesthetic to maintain the authenticity and integrity of such structures, thus, MICP as a nature-inspired method, has been proposed to repair historic stone buildings (Ortega-Villamagua et al., 2020; Ortega-Morales and Gaylarde, 2021). For soil stabilization, extensive studies have proposed this technique as an eco-friendly solution. Very recently, the ureolytic bacteria isolated from Hokkaido was used successfully *in situ* for the stabilization of expressway slopes of Hokkaido, Japan (Gowthaman et al., 2023). There are still some challenges faced by microorganisms, which need to be solved. Of particular concern is that mainstream biocementation research employs commercially available bacteria (e.g., *Sporosarcina* sp., *Bacillus* sp.) due to their high performance, which may lead to potential biohazard (Zhu and Dittrich, 2016; Portugal et al., 2020). Nevertheless, no comprehensive risk evaluation system has not been established for this issue yet, and general strategies are to utilize native strains and control the bacteria population being applied (Portugal et al., 2020). A set of researchers continuously explores isolates either from target soils or from possible sites within the zones of regulatory acceptance. Much isolation work has been focusing on soils, among which some studies have tried isolating bacteria from organic soils and applied them to stabilize local organic soils (Gowthaman et al., 2019; Gowthaman et al., 2023). Other researchers have isolated ureolytic bacteria from marine sediment for stabilization of the methane hydrate-bearing layer (Hata et al., 2020), from cave environments to seeking for calcifying strains (Omoregie et al., 2019; Jurado et al., 2022), from beach sand for coastal erosion prevention (Danjo and Kawasaki, 2016), *etc.* On the other hand, compared to the favorable conditions in laboratory-scale experiments, field-scale applications are subject to constant changes and interactions with local microbial communities. As a result, applied bacteria may fail to achieve the desired outcomes (Zhu and Dittrich, 2016).

What has been mentioned above highlights the need to address these issues to establish a more flexible technique aiming for application to various soils. Most of the researchers augmented an alkalophilic ureolytic bacteria, to produce calcium carbonate in laboratory-scale MICP studies. It is worth mentioning, however, for the borderless implementation of MICP, more options for the microorganisms with desirable metabolic capabilities must be considered, and this research work has made some efforts to overcome these obstacles by seeking solutions from microorganisms. Extensive research has grown up around the topic of bioaerosols in the built environment since it is one of the important risk factors associated with disease transmission (Prussin and Marr, 2015; Fujiyoshi et al., 2017; Chen et al., 2020). Up to now, much of the research work has been descriptive in exploring airborne bacteria, especially human pathogens, in terms of distribution, diversity, and characteristics, while there have been few attempts to assess their role in engineering applications (Gandolfi et al., 2013; Smets et al., 2016; Ruiz-Gil et al., 2020). Originally, airborne bacteria were emitted from common sources such as soil, rocks, plants, animals, and water in ecosystems by external forces. Air is usually described as a harsh environment that kills microbes by UV radiation, desiccation, starvation, *etc.* For instance, one result of evolutionary strategies is that some bacteria produce pigments to protect themselves from UV light exposure (Fröhlich-Nowoisky et al., 2016). Therefore, air could also be considered as a filtration system that exerts selective pressure to these wanderers, which leads to the survival of resilient species and the death of fragile ones. Selecting bacteria with high resilience to harsh environments provides a solution to the survivability issue. On the other hand, since airborne bacteria are ubiquitous, there is less concern about biohazard caused by the strains, and they should be more acceptable to the public compared to the exogenous strains.

The ureolysis is the most energy-efficient pathway of MICP (Dejong et al., 2013), and that has been the primary focus of geotechnical researchers to date. The process involves using the ureolytic bacteria to produce cementitious calcium carbonate minerals at soil contacts and within pores, resulting in reduced hydraulic conductivity and enhanced stiffness. This study aims to explore ureolytic airborne bacteria in an easily accessible elevation and investigates whether they could be effectively applied in MICP for soil improvement. Airborne bacteria of this study were collected from outdoor air at several sites around Sapporo City, Hokkaido using an air sampler that was designed for monitoring environmental hygiene. Isolates standing out from several rounds of screening were mainly evaluated by their capability of carbonate precipitation, growth pattern, urease activity, and MICP efficiency in the sand column test. The excellent efficiency of precipitation induced by two candidates confirmed the promising applicability of airborne bacteria toward biocementation. It is worth mentioning that this is the first study to make an original contribution to exploring resilient bacteria for MICP application from the air, the indispensable yet sometimes disregarded existence.

## 2 Material and methods

### 2.1 Sampling sites and sampling method

**FIGURE 1:**
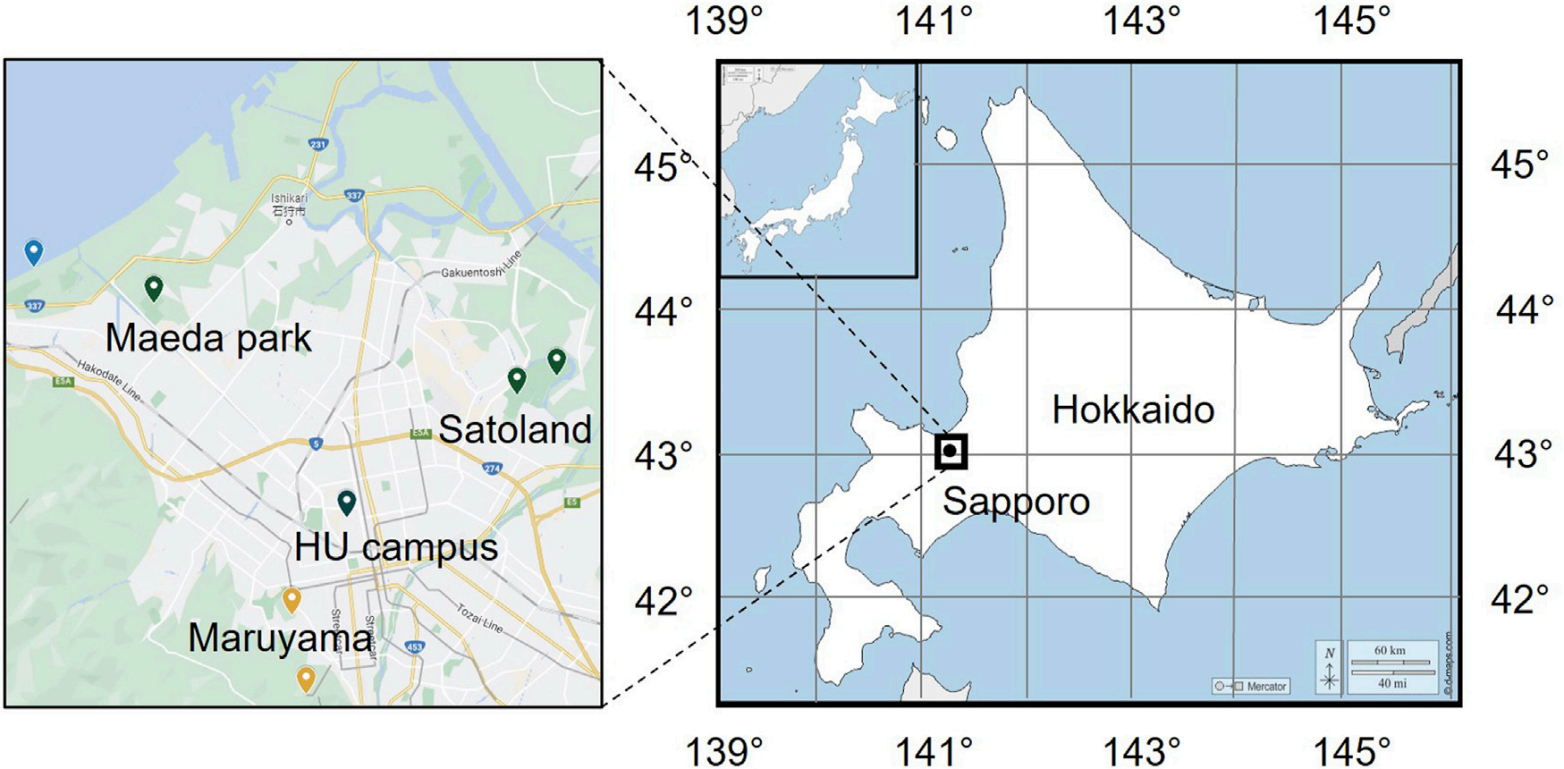
Location of investigation area and sampling. Earth maps were obtained from Google Map and the open access site d-maps.com (https://d-maps.com/pays.php?num_pay=515&lang=en).

**FIGURE 2:**
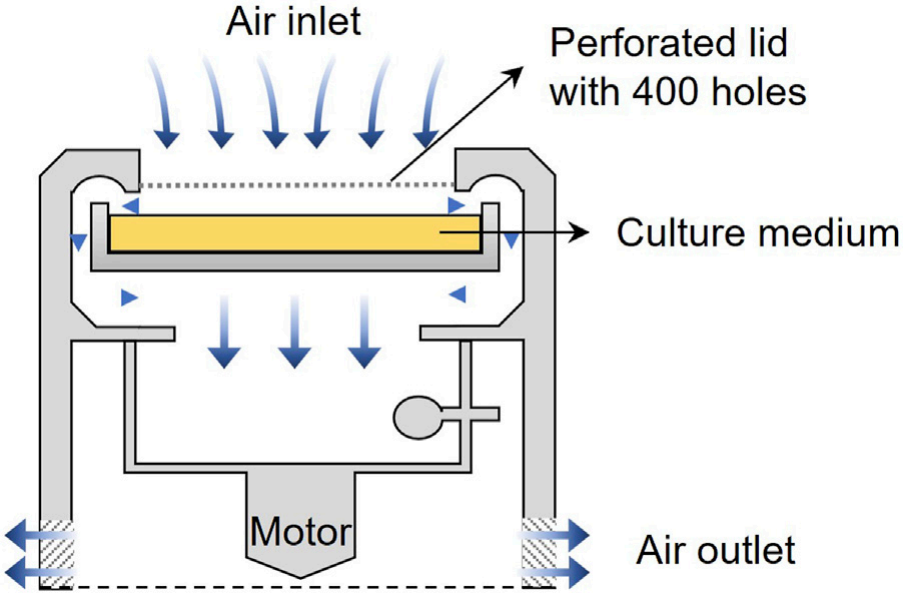
Sampling mechanism of air sampler MAS-100 Eco^®^.

The sampling sites in this study are located in some urban terrestrial areas of Sapporo, Hokkaido, Japan (see in Figure 1). Hokkaido, a cold region with an annual mean temperature of about 10°C, is located at the northern tip of Japan and surrounded by the Pacific Ocean, the Sea of Japan, and the Sea of Okhotskb. Sapporo is the capital city, with a low population density of 67 persons/km^2^ (MLIT of Japan). Table 1 presents the details of sampling sites. These sites are chosen due to the dense vegetation, as it has been reported that the leaf surface (4 times larger than that of the ground surface) is a large emission source of microorganisms (Després et al., 2012). The sampling was carried out on sunny days in the fall season of 2022. The temperature was mostly in the range of 15°C–25°C and the average wind speed is about 6.33 km/h. Based on the data provided by Japan Atmospheric Environmental Regional Observation System and Sapporo atmospheric environment observation data breaking system, the average air quality in the sampling month was good (PM2.5: 25–50 μg/m^3^; PM10: 0–25 μg/m^3^; O_3_: 25–50 ppm; NO_2_: 0–25 ppm; SO_2_: 0–25 ppm; CO: 0–25 ppm). An air sampler (MAS-100 Eco^®^, Merc Millipore) that is designed for monitoring environmental hygiene was used to collect airborne bacteria at an airflow of 100 L/min (±4%) for 500 s per sampling (see the mechanism in Figure 2). The culture medium NH_4_-YE (ATCC 1376) consists of 15.75 g/L of Tris-aminomethane, 10 g/L of ammonium sulfate, 20 g/L of yeast extract, and 20 g/L of agar. Before sampling, the perforated lid was cleaned with ethanol for disinfection to reduce contamination. After each sampling, the samples were sealed with PVC tape and stored in sterile Ziplock bags temporarily. All samples were then incubated at 30°C for 48 h before the isolation and screening process.

**TABLE 1:** Information of sampling sites.

Location	Latitude (N)	Longitude (E)	Altitude	Note
Hokkaido University (HU)	43°4′28″-43°4′44″	141°19′59″-141°20′15″	10–20 m	Rice field, dairy farm, sheep pen, forest, lawn
Maruyama (MY)	43°2′5″-43°3′9″	141°18′31″-141°18′59″	60–220 m	Mountain, forest
Maeda (MD)	43°8′39″- 43°8′52″	141°15′11″-141°15′23″	10 m	Forest Park
Satoland (SL)	43°6′58″-43°7′10″	141°24′48″-141°25′3″	10 m	Amusement Park with penned animals

### 2.2 Isolation and screening method

**FIGURE 3:**
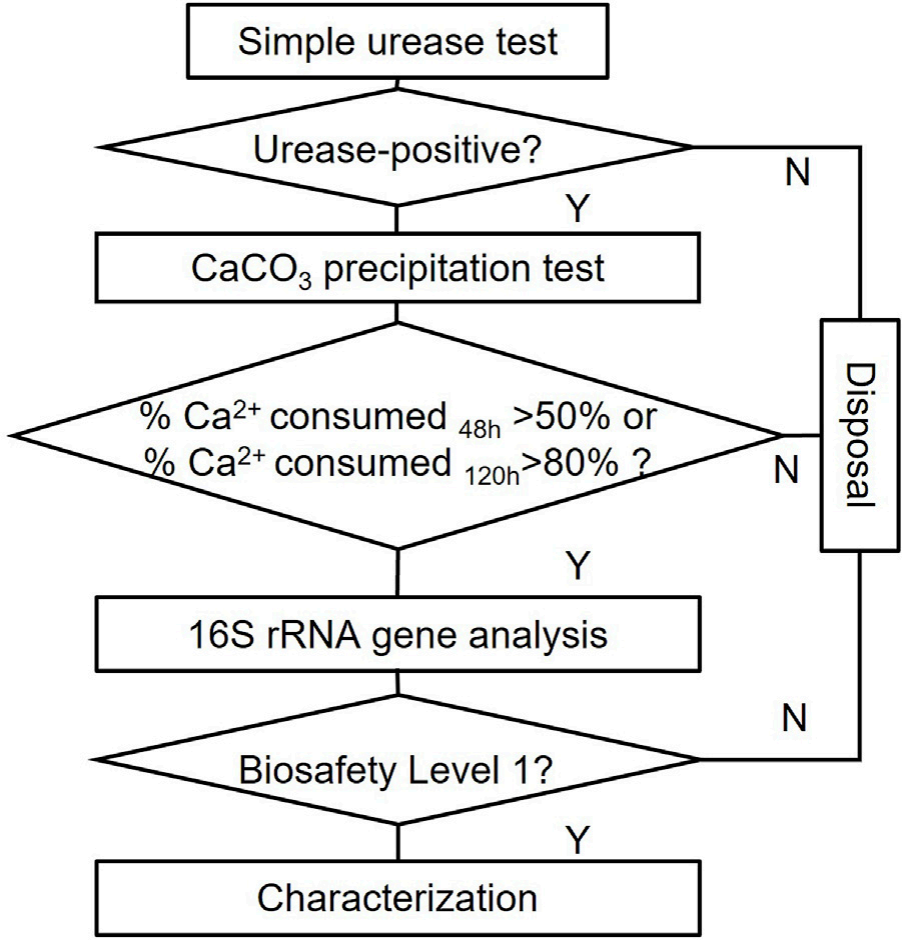
Isolation and screening flow of this study.

Easily visible colonies were inoculated on new NH_4_-YE agar plates. By streaking, the purified single colonies after 24 h were ready for further tests. The screening procedures could be simplified as three rounds of screening, as seen in Figure 3.

#### 2.2.1 Simple urease test

To begin this process, a simple and rapid test using cresol red is adopted to identify ureolytic bacteria efficiently. During the process of urea hydrolysis, the pH of the solution increases over time. A simple urease activity test using cresol-red could confirm urease activity by a qualitative observation of the color change from yellow to purple, indicating an increase in pH. 100 mL test water solution consists of 2.5 g of urea and 2 mL of 4% cresol-red ethanol solution. The bacteria were added to the testing solution, then incubated at 45°C for 2 h. Species that changed the color into purple were identified as urease activity positive (Danjo and Kawasaki, 2016).

#### 2.2.2 Calcium carbonate precipitation test

Bacteria were prepared in liquid culture and evaluated in calcium carbonate precipitation tests under second-round screening which also rules out some bacteria that fake the first-round test by other metabolic activities. The tests were conducted at room temperature to confirm the bacterial ability to induce calcium carbonate precipitation. For each test tube, 2 mL of bacterial main culture was added into 8 mL of 0.5 mol/L of CaCl_2_ and urea solution. The calcium meter used in this measurement is a compact Ca^2+^ meter B-751 (HORIBA, Ltd., Kyoto, Japan), with a measurement range from 4 to 9,900 ppm. For each sampling, 100 µL of the solution was taken and diluted before measurement. In an attempt to select both fast and slow precipitators, two lines were set up to select potential candidates.

#### 2.2.3 16S rRNA gene analysis

The DNA of selected isolates was extracted using MightyPrep reagent for DNA (TAKARA Bio Inc.) following the instructions provided by the manufacturer. The PCR components include 25 µL of Primestar Max (TAKARA PrimeSTAR Max DNA Polymerase), 1.5 µL of forward primer 9F (5′-GAG​TTT​GAT​CCT​GGC​TCA​G-3′), 1.5 µL of reverse primer 1541R (5′ AAG​GAG​GTG​ATC​CAG​CC-3′), 1 µL of DNA template, and 21 µL of sterile distilled water. The thermal cycler (T100 Bio-Rad Laboratories, Inc.) started the PCR with a 30-s initial denaturation at 94°C, and the thermal cycling process consisted of 30 cycles of 10-s denaturation at 98°C, 5-s annealing at 55°C and 8-s extension at 72°C. Agarose gel electrophoresis was then conducted to visualize and purify the PCR amplicons. Bands that appear around 1,500 base pairs were cut from the gel and DNA was extracted from the gel using FastGene Gel/PCR Extraction Kit (NIPPON Genetics Co., Ltd.). Six primers were used in the sequence adjustment process: 9F, 515F (5′-GTG​CCA​GCA​GCC​GCG​GT-3′), 1099F (5′-GCA​ACG​AGC​GCA​ACC​C-3′), 1541R, 1115R (5′-AGG​GTT​GCG​CTC​GTT​G-3′), and 536R (5′-GTA​TTA​CCG​CGG​CTG​CTG-3′). Adjusted samples were then sent to Eurofins Japan to analyze the sequence. Finally, BLAST (Basic Local Alignment Search Tool) analysis was performed using the database DB-BA17.0 (Techno Suruga Laboratory, Japan). Under Japanese laws as to microorganism utilization, it is necessary to examine the bio-safety level (BSL) of bacteria before applying the microorganism to a field scale. The isolates confirmed as Biosafety Level one species were stored at −80°C in glycerol stock for further characterization.

### 2.3 Characterization of selected strains

Bacteria were inoculated from an agar plate to a 5 mL-pre-culture test tube and cultured for 24 h. For 100 mL of the main-culture, 1 mL of pre-culture was inoculated, then incubated in a shaker at 160 rpm. Quantitative measurement of bacterial population was conducted using a spectrophotometer, which tests the concentration of a solution by measuring its optical density of a specific wavelength of light (600 nm). A quick determination of urease activity is using a conductivity meter to monitor the conductivity changes with time (Whiffin et al., 2007). This measurement uses a compact EC meter (LAQUAtwin EC-33, HORIBA, Ltd., Kyoto, Japan). With 3-point calibration, the accuracy is ±2% full scale. For testing, 0.5 mL of bacterial culture is added into a beaker with 50 mL of 0.1 mol/L urea solution placed on a stirrer in a water bath (Thermal Robo TR Series, AS ONE Corporation) at 25°C. One sampling of 0.12 mL is taken from the bacteria-urea solution and measured at 5-min intervals (0, 5, 10, 15-min).

### 2.4 Sand solidification test and evaluations

Before the MICP treatment, duplicate cases packed 60 g of coarse silica sand (97.65% of SiO_2_, 0.89% of Al_2_O_3_, 0.43% of Fe_2_O_3_, 0.18% of K_2_O, 0.11% of MgO, and 0.11% of Na_2_O) with a mean grain size of 0.6 mm uniformly in a 50 mL syringe. The method of solidification test is a 14-day treatment that consists of two-phase injections: 12 mL of bacteria culture at stationary stage per week and 12 mL of 0.5 M CaCl_2_ and urea (with 1.5 g/L nutrient broth) per day (Cheng and Cord-Ruwisch, 2014; Gowthaman et al., 2019). Before measurement of unconfined compressive strength (UCS), MICP-treated samples were rinsed with distilled water to remove soluble salts and then oven-dried at 60°C for 48 h (Ahenkorah et al., 2021). The needle penetration test adopted to estimate the UCS is recommended by International Society for Rock Mechanics (ISRM) for testing soft rocks and cemented soil. There is a regression relationship (Eq. 1) between the penetration depth and the penetration resistance, which was developed by testing 50 cemented soil samples and 114 natural soft rock samples (Azulay et al., 2018).

$$\log\left(y\right)=0.987\log\left(x\right)+2.621$$ (1)

where y is the UCS; x is the penetration gradient (N/mm) determined by the penetration depth and penetration resistance.

The CaCO_3_ content, as one of the most important factors that evaluate the MICP efficiency, was measured as the gas pressure produced when samples react with acids using a device developed by Fukue et al. (1999). Details were described in the previous study (Chen et al., 2021). Oven-dried samples were then observed under Miniscope TM 3000 (Hitachi, Tokyo, Japan) and the X-ray diffraction pattern was analyzed using MultiFlex (Rigaku Co., Ltd., Tokyo, Japan) to demonstrate the polymorphs of induced CaCO_3_. The measurements were conducted using Cu-Kα radiation at 40 kV, 40 mA, 6.5°/min.

## 3 Results

### 3.1 Urease-positive percent by sampling sites

**FIGURE 4:**
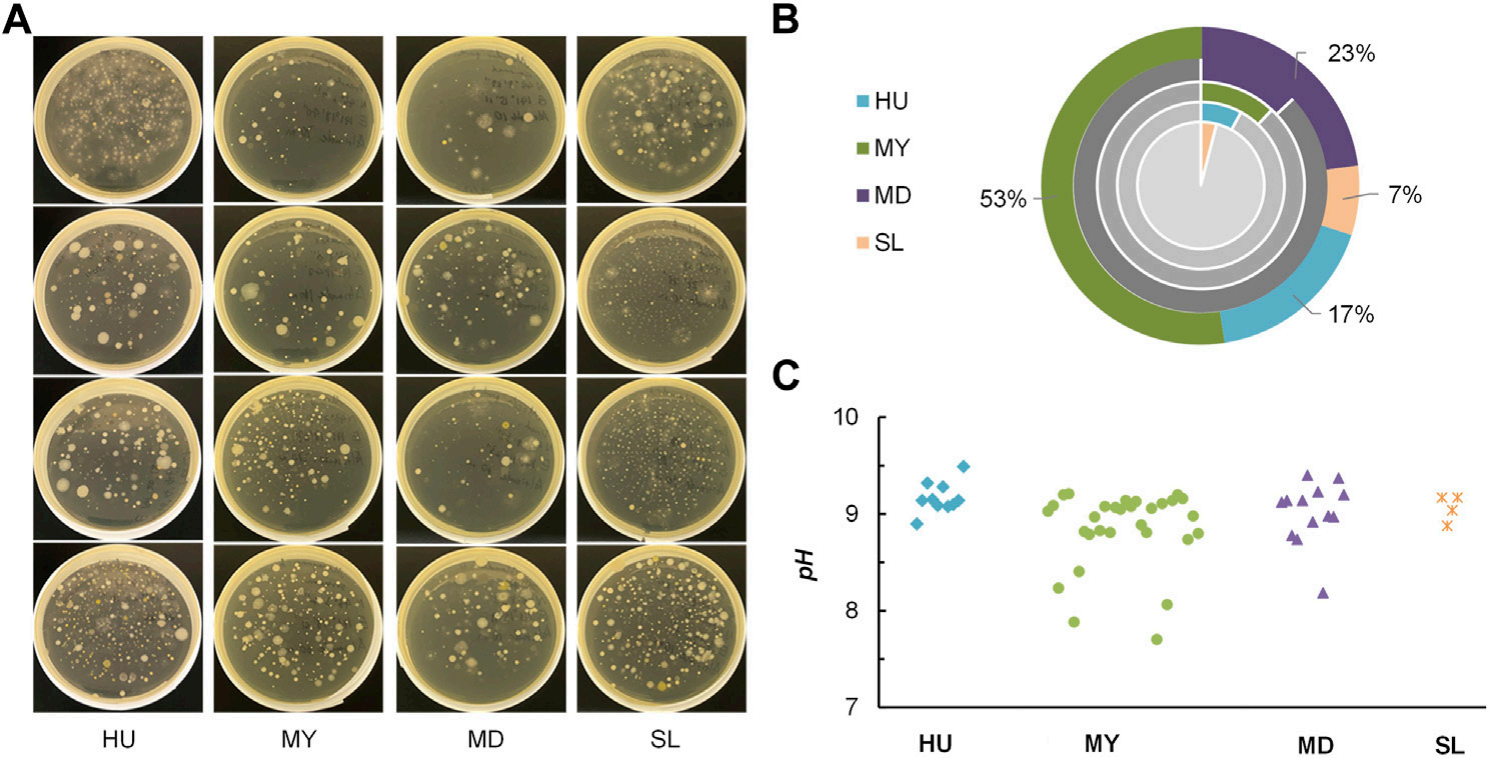
**(A)** Representative images of samples from four sites; **(B)** percent of urease-positive isolates from each site (pie charts: grey-negative, color-positive), composition of 57 selected isolates (doughnut chart) **(C)** pH of cresol red-urea solution in simple urease tests after 2 h.

In total, 22 no. of samples were obtained, with 8, 6, 4, 4 isolates from Hokkaido University (HU), Maruyama (MY), Maeda (MD), and Satoland (SL), respectively. Figure 4A provides images of representative airborne bacteria sampled from four sites. It can be seen that there are some notable differences between these samples. For instance, more undesired fungi colonies grew on plates sampled from sites with penned animals compared to that on other samples. Among 577 tested colonies, 57 were identified as urease positive by simple urease tests. The urease-positive percent by sites is summarized in the pie chart Figure 4B. The pH of cresol red-urea solution was measured after a 2-h incubation at 45°C, as seen in Figure 4C. In general, more ureolytic strains were found in sites with more dense vegetation. Totally, 57 isolates (10 from HU, 30 from MY, 13 from MD, and 4 from SL) were selected and examined in the calcium carbonate precipitation tests.

### 3.2 Monitoring of calcium carbonate precipitation

**FIGURE 5:**
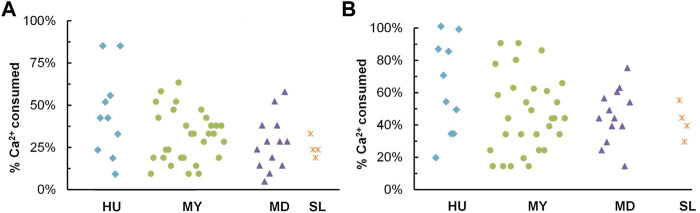
Calcium carbonate precipitation test. Percent of calcium ions consumed **(A)** after 48 h; **(B)** after 120 h.

Figures 5A, B below presents percents of calcium ions consumed by 57 isolates after 48-h and 120-h incubation. On average, isolates from HU were shown to have a carbonate precipitation capability in the test, followed by MY, MD, and SL isolates. Isolates that precipitated more than 50% calcium ions after 48-h incubation and that precipitated more than 80% calcium ions after 120-h incubation were selected as potential candidates for MICP application. Ultimately, 5 isolates from HU, 5 isolates from MY, and 2 isolates from MD were identified by 16S rRNA gene analysis and then characterized in the following tests.

### 3.3 Bacterial identification

The sampling method based on cultivation generally finds more Gram-positive strains since Gram-negative bacteria are more likely to lose their cultivability in the air (Després et al., 2012). The findings in this study are no exception. Table 2 summarizes basic information of 12 isolates that all belong to Biosafety Level 1, including one opportunistic pathogen. Most of the sequences matched with known species with their identity as high as 99%. The nucleotide sequences of 12 isolates have been deposited in DDBJ database, and the accession numbers can be found in Supplementary Table S1. According to the information provided by Microbe Atlas Project (MAP, 2023), the closest strains were mostly previously from origins such as soil and animals. What stands out among isolates are RII-2 and FU3 isolated from a rice field and a forest on campus, which were identified as strains from *Lederbergia* genus that was reclassified from *Bacillus* genus by Gupta et al. (2020). It should be mentioned here that the first strain RII-2, *Lederbergia lenta* (synonym: *Bacillus lentus*), was reclassified from *Bacillus* genus that consists of many ureolytic strains. The latter one is possibly a new species in *Lederbergia* genus since it has a similarity of 96.6% to its closest species. These two isolates were able to precipitate carbonate in a short period, which is much more active than other isolates, therefore, they are proceeded to the characterization tests.

**TABLE 2:** Summary of the taxonomy of twelve isolates.

Isolate ID	Isolation source	Closest taxonomy				Global distribution (microbeatlas) samples from different sources			
		Genus_Species	Family	Identity (%)	Accession	Aquatic	Soil	Animal	Plant
RII-2	Rice paddy (HU)	*Lederbergia lenta*	*Bacillaceae*	98.5	AB021189	15	300	167	12
FU3	Forest (HU)	*Lederbergia* sp.	*Bacillaceae*	96.6	AJ535638	93	412	390	82
FU17		*Glutamicibacter soli*	*Micrococcaceae*	99.4	EF660748	82	74	419	45
FU4^a^		*Staphylococcus saprophyticus*	*Staphylococcaceae*	99.9	AP008934	1,554	930	23,674	763
CH3	Cow house (HU)	*Glutamicibacter protophormiae*	*Micrococcaceae*	99.2	X80745	1	33	39	4
MY5-21	Summit-220 m	*Glutamicibacter mysorens*	*Micrococcaceae*	99.2	AJ639831	29	26	63	9
MY3-21	Low brush −150 m	*Glutamicibacter arilaitensis*	*Micrococcaceae*	99.1	AJ609628	149	92	507	69
MY3-15		*Lysinibacillus parviboronicapiens*	*Bacillaceae*	99.1	AB300598	8	122	9	3
MY2-9	Low brush- 110 m	*Sporosarcina globispora*	*Planococcaceae*	98.6	X68415	2,194	10,719	3,706	1805
MY1-15	Low brush −70 m	*Glutamicibacter soli*	*Micrococcaceae*	99.4	EF660748	82	74	419	45
MD4-29	Maple forest	*Sporosarcina koreensis*	*Planococcaceae*	99.6	DQ073393	-	-	-	-
MD4-5		*Psychrobacillus lasiicapitis*	*Bacillaceae*	99.3	KP219721	3	15	3	1

^a^ Opportunistic pathogen.

**FIGURE 6:**
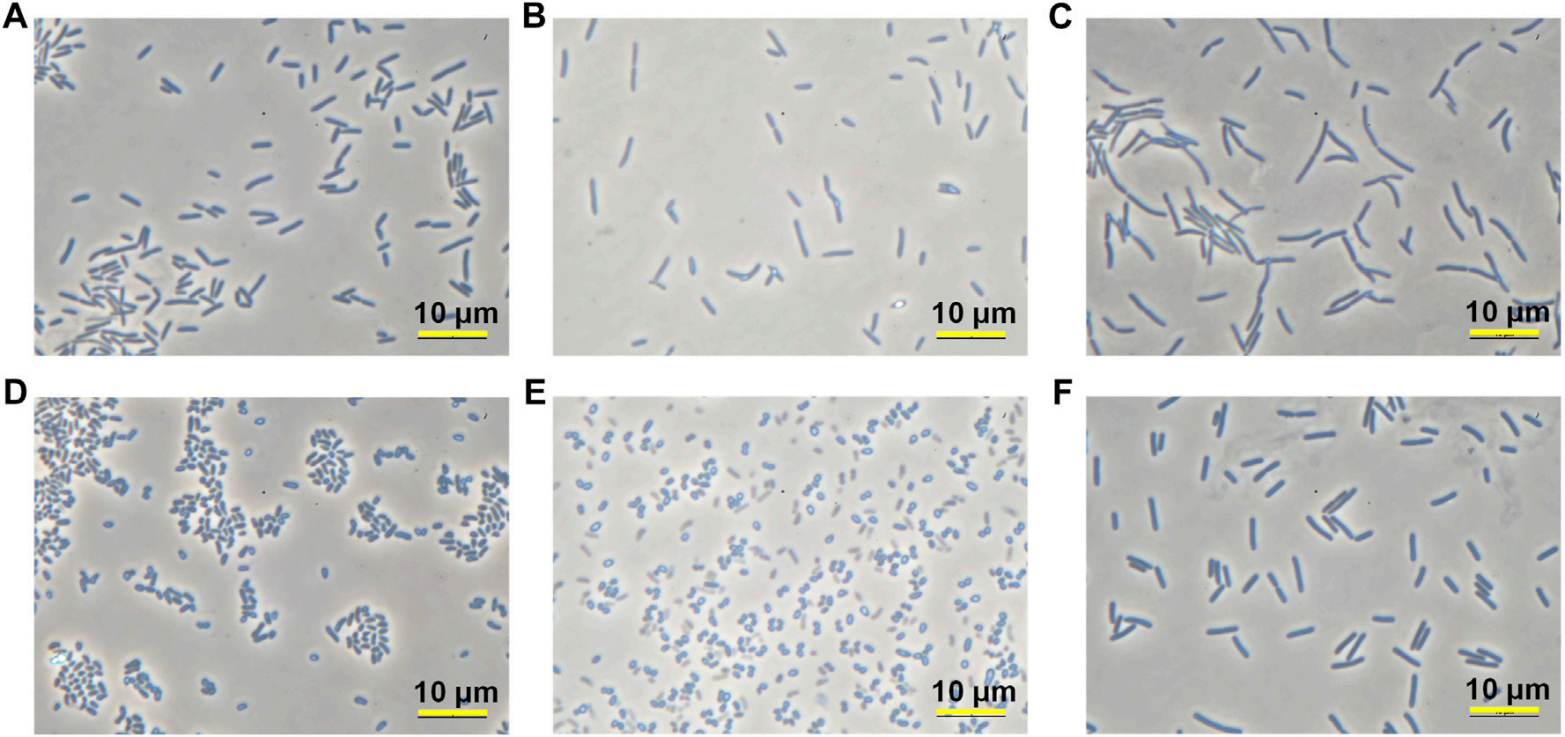
Microscope images of four isolates after certain period of cultivation: **(A,B)** FU3 after 24 h and 120 h; **(C)** RII-2 after 24 h; **(D,E)** MY3-21 after 24 h and 120 h; **(F)** MY2-9 after 24 h.

Twelve isolates are from *Firmicutes* (7 isolates) and *Actinobacteria* (5 isolates) which are two of four major phyla that contain most of the culturable species. In particular, five strains from *Actinobacteria* phylum were found to belong to *Glutamicibacter* genus that was reclassified from *Arthrobacter* genus (Busse, 2016). Many airborne bacteria survive in the air in dormant forms and reactivate themselves when the environment becomes favorable to them. Since *Glutamicibacter* is not a spore-former, they should be resilient against desiccation, starvation, and UV radiation. As many bioremediation reports have found that strains from this genus could survive a long period and changing conditions (Alvarez et al., 2017; He et al., 2017; Chen et al., 2022), it would be an advantage if they can be applied to MICP research. Therefore, MY3-21, one *Glutamicibacter* species showing good precipitation capacity was selected and characterized in the following tests. On the other hand, MY2-9 was selected and examined as a representative strain of *Sporosarcina* genus among 12 isolates. Microscope images (Microscope BBX50F4, Olympus, Japan) in Figure 6 show the morphology features of selected four isolates. FU3 and RII-2 are two typical rods, while the former (0.9–1.0 × 1.5–5.0 μm) is shorter than the latter (0.9–1.0 × 2.5–7.0 μm). MY2-9 has a similar cell shape and size (0.9–1.0 × 4.0–7.0 μm) as RII-2. MY3-21 shows a pleomorphism similar to *Arthrobacter* genus, changing their shape from rods to cocci (1.0–1.1 × 1.0–2.5 μm) during cultivation.

### 3.4 Growth curve of selected strains

**FIGURE 7:**
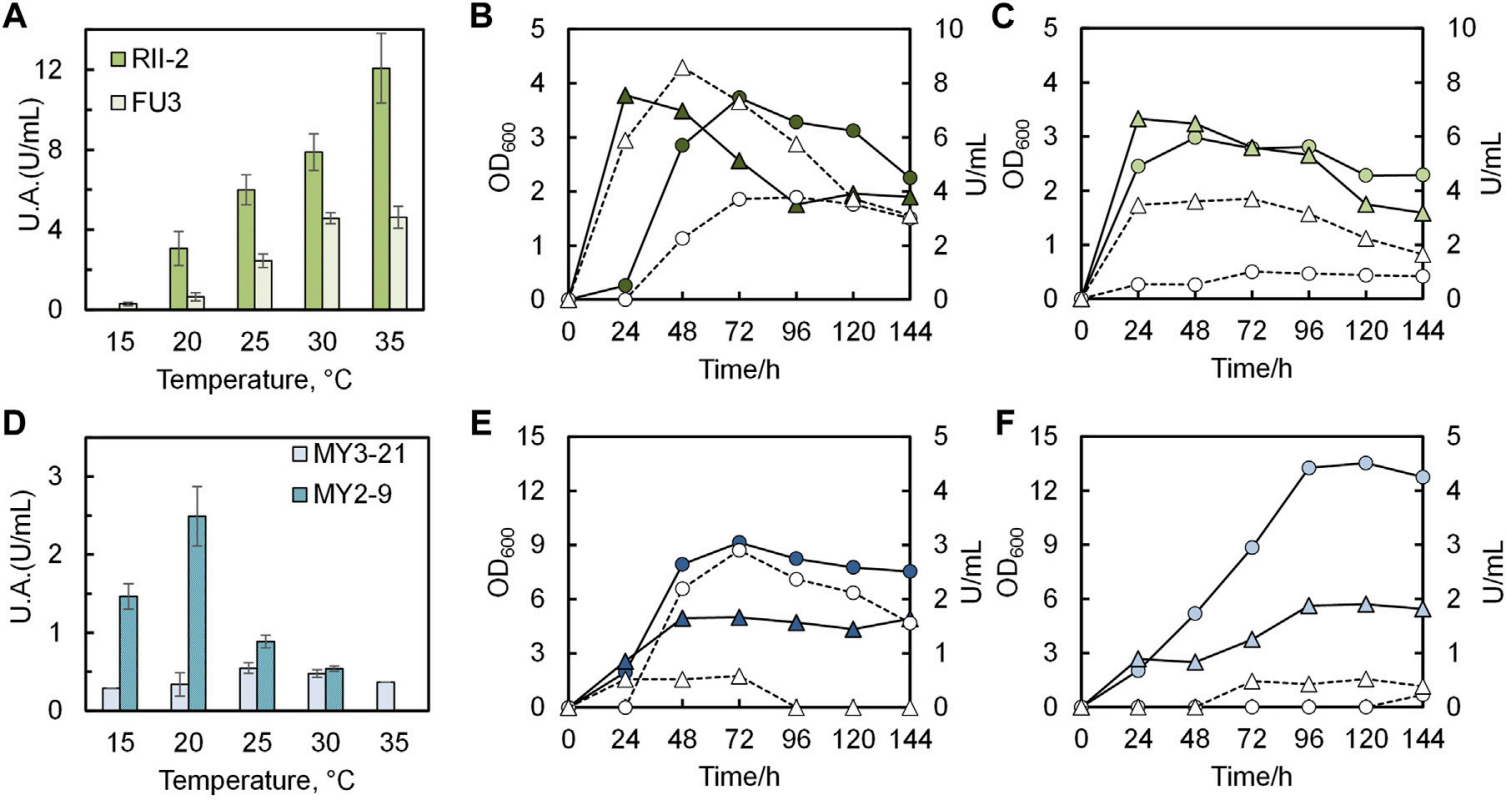
Temperature dependence of four selected isolates. Peak U.A. under 15°C–35°C of **(A)** RII-2 and FU3; **(D)** MY2-9 and MY3-21. Growth and activity changes with time under 20°C (OD600-●; U/mL-○) and 30°C (OD600-▲; U/mL-∆) of **(B)** RII-2; **(C)** FU3; **(E)** MY2-9; **(F)** MY3-21.

Four isolates that showed excellent performance at precipitation tests were characterized by urease activity tests under varying temperatures, ranging from 15°C to 35°C. At the same time, the growth curve of each strain was monitored every 24 h. As seen in Figure 7A, there is a rapid increase of growth rate and urease activity with increasing temperature both in the case of RII-2 and FU3. And the activity of the former is likely to continue increasing, whereas that of the latter will decline when the temperature exceeds 35°C. Figures 7B, C provides the growth curve and urease activity of two strains under 20°C and 30°C, showing a peak activity after 24-h or 48-h cultivation and a steady decline after their stationary phase. In general, RII-2 and FU3 might be two potential candidates for MICP in a tropical temperate region. The temperature dependence of two strains from Maruyama Mountain is illustrated in Figure 7D in which MY3-21 and MY2-9 show notable differences compared to the previous two strains in several respects, such as the growth pattern and activity under varying temperatures (see Figures 7E, F). Although both of them tend to have a higher growth rate under low temperatures, the activities have shown optima at 25°C and 20°C. Interestingly, in reported studies on the closest strain of MY2-9 *Sporosarcina globispora*, it is often classified as a psychrophile (Wang et al., 2015). It is unique among 12 isolates that are mostly reported as common mesophiles. Ideally, this strain could be a good precipitator for cold regions. On the other hand, what is unexpected in this figure is that the MY3-21 showed a very limited activity although it seems to be a good carbonate precipitator in previous tests. Possible explanations are that the cell membrane composition of *Glutamicibacter* sp. (glutamic acid in the peptidoglycan interpeptide bridge) might contain certain groups that facilitate the precipitation process and species of this genus might have a good tolerance toward high concentrations of calcium source and urea. It should be noted that some amino acid groups influence precipitation (Nawarathna et al., 2021). In this case, this strain could be a potential candidate for MICP application. In this baseline study, the feasibility of applying airborne bacteria was confirmed by small-scale solidification tests under room temperature using two strains with the highest activity among isolates, RII-2 and FU3.

### 3.5 Evaluation of MICP-treated samples

**FIGURE 8:**
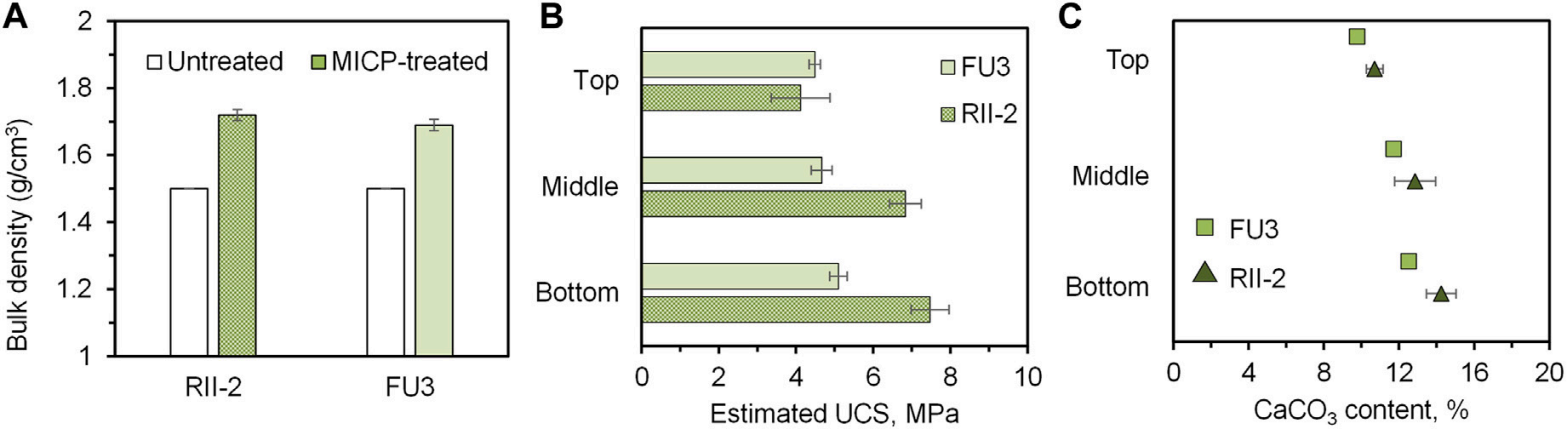
Evaluation of MICP-treated samples. **(A)** Before-after comparison of bulk density; **(B)** estimated unconfined compressive strength and **(C)** estimated calcium carbonate content of top, middle, and bottom of treated samples.

The treated samples were evaluated both mechanically and chemically. The bulk density changes in samples after treatment were shown in Figure 8A, revealing a density increment of about 12% in two cases. The results of the estimated UCS obtained from needle penetration tests are presented in Figure 8B. What can be seen in this figure is the increase of strength with depth, which results from a general pattern of calcium carbonate distribution in coarse sand columns. As the bacteria permeate through the coarse sand column from the sample surface to the bottom, some cells are trapped along the way down while others go down with effluent. The calcium carbonate content results seen in Figure 8C were consistent with the UCS tendency, increasing the carbonate content led to an improvement in strength. It is worth noting that approximately 90% provided calcium ions were precipitated in the case of RII-2 and FU3 precipitated about 80% of that, indicating a highly efficient utilization of reagents for MICP. From the distribution of the calcium carbonate precipitation and the corresponding strength improvement, FU3 seems to be contributing to a more uniform cementation than RII-2, although it has a lower activity compared to RII-2. From the microscope images in Figure 6, it can be seen that FU3 is smaller in cell size, however, it seems that more clusters are forming compared to the RII-2 arrangement. Extracellular polymeric substances of bacteria have been found to affect the formation of calcium carbonate in terms of morphology and structure (Azulay et al., 2018). Differences in biopolymers at the bacterial surface may contribute to a higher retention rate at the upper layer of sand columns. Since FU3 is probably a new species in *Lederbergia* genus, its characteristics are still unknown. Therefore, more investigation is needed to understand the mechanism behind this finding.

**FIGURE 9:**
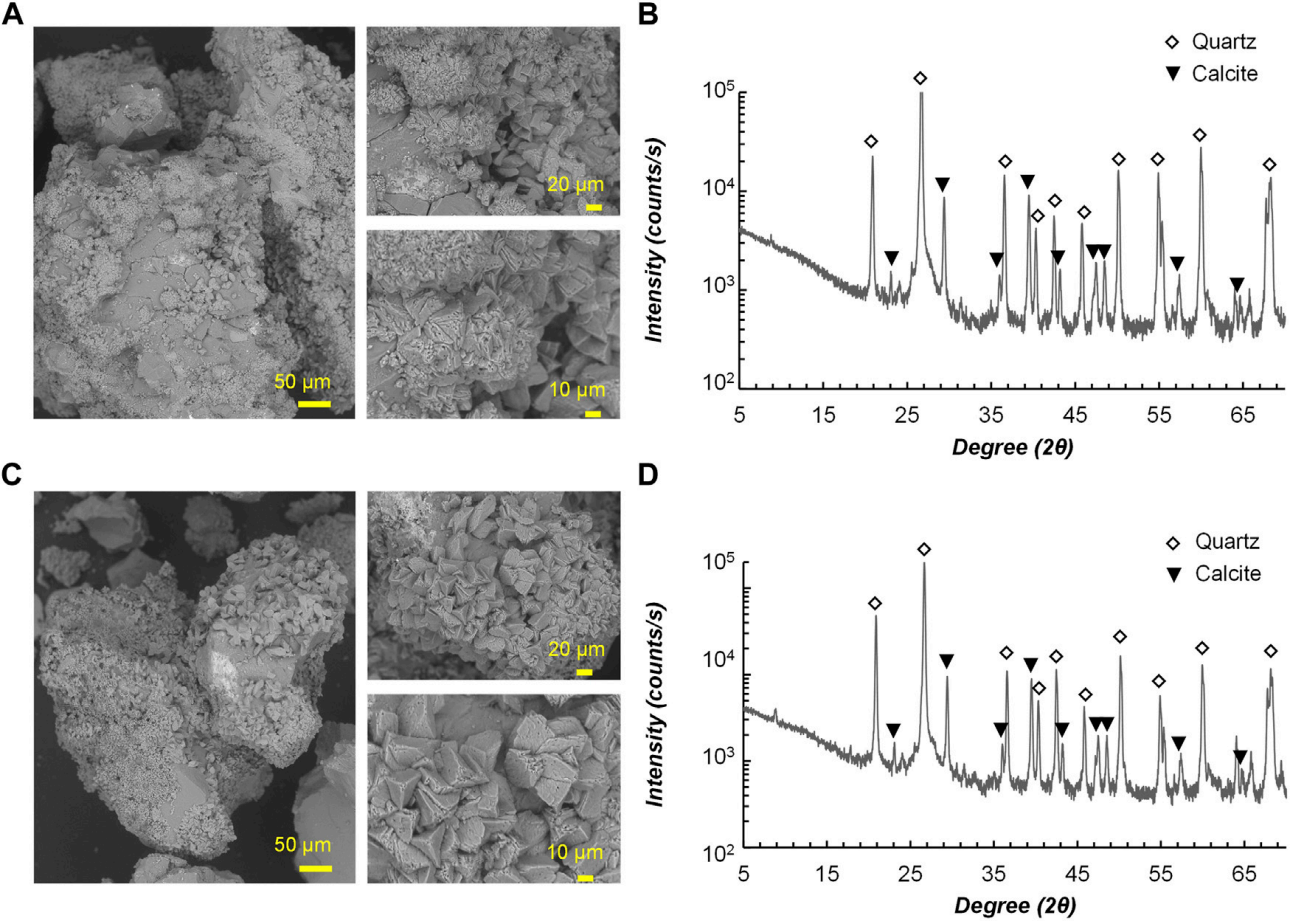
Scanning electron microscope images of precipitates for **(A)** RII-2; **(C)** FU3. X-ray diffraction analysis results for **(B)** RII-2; **(D)** FU3.

Figure 9 illustrates the MICP-treated samples under scanning electron microscope observation (Figures 9A–C) and analysis of XRD (Figures 9B–D). From the SEM images, there is no significant difference found between the precipitates induced by the two strains in terms of morphology, and mostly the size of precipitates fell into the range of 10–20 µm. It can be inferred from the tetrahedral form that the precipitates are calcite, which was further confirmed by the peaks in XRD patterns. Khanjani et al. (2021) investigated the controlling factors of microbial-induced CaCO_3_ precipitation using *Sporosarcina pasteurii*, from which the results found that the morphology of CaCO_3_ changes with increasing Ca^2+^ and urea concentrations, and the vaterite formation dominates at early stages and transforms to calcite after a certain period under aqueous conditions. In this study, the transformation of calcium carbonate phases might have been done during the treatment, which explains why calcite is the only calcium carbonate form identified.

## 4 Discussion

**FIGURE 10:**
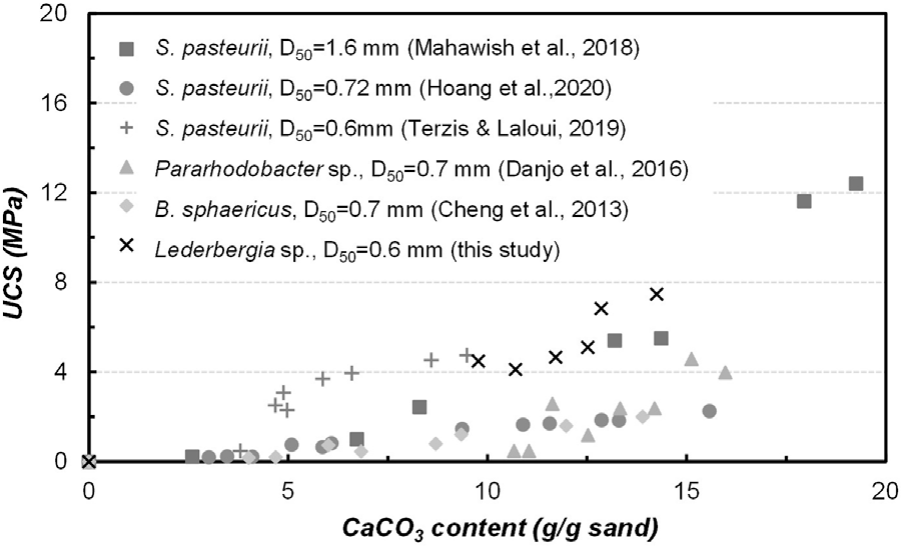
Relationship between calcium carbonate content with UCS-a comparison with published studies on coarse sand solidification by MICP.

The correlation between unconfined compressive strength with calcium carbonate content is usually discussed as important evidence of MICP efficiency, which could vary to some degree depending on the grain size distribution of tested sands, applied bacterial species, and treatment design. In this study, a comparison seen in Figure 10 was made to show the difference between studies on coarse sand with different species applied (Cheng et al., 2013; Danjo and Kawasaki, 2016; Mahawish et al., 2018; Terzis and Laloui, 2019; Hoang et al., 2020). As a, namely, gold standard of ureolytic bacteria, *S. pasteurii* has been the preferred strain for most MICP researchers due to its excellent performance. What can be seen in this figure is that the *Lederbergia* species in this study is comparable in bonding efficiency to that of *S. pasteurii*, which is a remarkable outcome that confirms the applicability of these two strains. What was found in our previous sand column solidification tests under the same experimental conditions (bacterial urease activity 3.5 U/mL) is that the bacterial performance began to decrease significantly after 5 days, leading to a relatively low calcium carbonate content and weak cementation effect (Chen et al., 2021). Although the bacteria showed similar urease activity under urease test condition, their performances in sand column solidification tests differ. Possibly, two *Lederbergia* strains have a better tolerance towards high concentration of calcium ions and ammonium and a higher survival rate under nutrient-deficiency environment. To confirm this postulation, further investigation is necessary for the subsequent stage of this research. Overall, these two strains could be good precipitators for MICP application in moderate climates.

Compared with soilborne bacteria, airborne bacteria are more likely to be resilient strains. It has been reported that they can survive desiccation, nutrient deficiency, temperature shifts, harmful UV radiation, *etc.*, which is possibly associated with DNA repair mechanisms, pigmentation, aggregation behavior, and adaptation to nutrient deficiency (Smets et al., 2016). Since the airborne bacteria usually withstand desiccation, it is highly possible that they could also survive in the process of freeze drying for obtaining a culture starter (Gratia et al., 2009). This would be an essential advantage over other bacteria with less resistance to desiccation when considering the production and application of ureolytic bacteria for MICP. Potential performance under nutrient deficiency conditions indicates the daily nutrient supply may not be necessary for the treatment, which might also contribute to a less cost of bacterial cultivation. On the other hand, when they are applied to regions with significant temperature differences between daytime and nighttime, they are more likely to survive and induce continuous precipitation. For example, MY2-9 which thrives at a wide range of temperatures and produces cold adaptive enzymes, might be able to perform stable precipitation under changeable temperatures. Specific airborne bacteria utilizing pigment as a protection from UV radiation can be applied to surface treatment without shading measurement, which would be good candidates for surface restoration of stone historic heritage. Several *Glutamicibacter* isolates in this study can produce yellow pigments. One of them pigments in a light-dependent manner as a protection strategy, which has been previously well-researched by Sumi et al. (2019). Overall, with many characteristics of great bio-technological significance, ureolytic airborne bacteria are supposed to be able to apply to MICP for various purposes.

### 4.1 Limitations of the current study and future prospects

As a baseline research work, this study mainly investigated the occurrence of ureolytic airborne bacteria in a specific region, screened and isolated potential candidates for MICP application, and evaluated the selected strains under a conventional treatment approach. However, to further demonstrate the advantages of airborne bacteria, examinations of the subsequent stage should be focusing on the survivability and adaptability issues under changeable conditions compared to commonly researched ureolytic strains. For instance, temperature conditions during the treatment should be designed to mimic the field environment, which is particularly important for MICP application in regions like Hokkaido. As natural soils have significant diversity in physical and chemical characteristics, whether the bacteria could survive and function normally to induce precipitation should be evaluated. In this study, two spore-forming strains with relatively high urease activity were evaluated in solidification tests under moderate temperatures, while non-spore formers among these ureolytic isolates should also be considered as potential candidates. On the one side, these fast precipitators function at certain temperature ranges, which could be a limitation to the applicable region. Non-spore formers isolated from this study all belong to *Glutamicibacter* genus, some of them were able to precipitate carbonate slowly but at a steady rate and survive for a long period. Therefore, these strains with great viability might contribute to continuous precipitation even after fast precipitators slow down and die off in the case of no significant negative effect resulting from interactions of strains. Ideally, the combination of strains with high urease activity and five *Glutamicibacter* sp. with relatively low activity but high survivability probably could contribute to an increased MICP efficiency. Furthermore, when dealing with problematic soils with complicated characteristics, modification of soil properties is usually necessary to provide the bacteria with a favorable environment to induce carbonate precipitation, which is a challenging task sometimes. A promising approach is to tackle the issue with multiple strains that play different roles to contribute to an effective MICP process.

### 4.2 Recommendations for further exploration of airborne bacteria

As a baseline study, the isolation method that used general media for ureolytic bacteria instead of selective media figured out the percentage of urease-positive strains in the air of four sampling sites, which made the screening method time-consuming and inefficient. Many factors influence the calcium carbonate precipitation in MICP process. From the aspect of ureolytic bacteria, not only the urease activity and carbonic anhydrase activity but also the compositions of bacterial cell surface and metabolites that they produce govern the formation of calcium carbonate (Ercole et al., 2007). Selective media with both urea and calcium sources added could quickly identify ureolytic bacteria by visualization of calcium carbonate on an agar plate. Most bacteria could tolerate urea concentrations up to 2% and could be inhibited by a higher concentration, but some ureolytic bacteria (e.g., *Sporosarcina ureae*) thrive with high concentrations of urea up to 8% (Madigan et al., 2021). Concentrations of urea and calcium sources could be set in a certain range depending on the target strains. On the other hand, sampling sites in this study were limited in certain topography conditions, other terrestrial or coastal environments should be investigated.

## 5 Conclusions

The present study explored airborne bacteria, often referred to as “microbial dark matter”, for resilient candidates for MICP application. Airborne bacteria were collected from four sites in an urban area and selected isolates were evaluated by a series of tests to confirm their MICP applicability. The findings provide new insights for isolation sources for ureolytic bacteria that are applicable to MICP. Based on the results, the following conclusions can be drawn.

• Urease-positive strains in cultivatable airborne bacteria collected from four sites are mostly less than 10%, and the isolates from sites with substrate sources tend to show higher activity than that from other sites.
• Among 57 urease-positives isolates, twelve of them were identified by gene analysis after two rounds of screening. About half of the identified isolates were spore-forming *Bacillus* relatives, and five non-spore-forming strains belong to *Glutamicibacter* genus.
• Bacterial growth pattern and urease activity of four selected isolates under 15°C–35°C cultivation showed that two high-activity *Lederbergia* strains thrive at relatively high temperatures, while two representatives of *Sporosarcina* and *Glutamicibacter* genera grow better at low temperatures.
• Sand column solidification tests using two *Lederbergia* strains confirmed the applicability of airborne bacteria for MICP, showing estimated UCS ranging from 4 to 8 MPa and precipitation of 80%–90% total calcium source.
• SEM and XRD analysis revealed that the tetrahedral precipitates induced by two *Lederbergia* strains are calcite, the most stable form of calcium carbonate.
• Overall, this study provides new insight into ubiquitous airborne bacteria for geotechnical engineering applications. Further investigation is needed to gain a more comprehensive understanding of their survivability and adaptability under changing conditions.

## Data Availability

The datasets presented in this study can be found in online repositories. The names of the repository and accession numbers can be found in the Supplementary Material. Further inquiries can be directed to the corresponding author.
