# Supplementary material for: Baseline investigation on soil solidification through biocementation using airborne bacteria
Source: Front Bioeng Biotechnol. 2023 Jun 14;11:1216171. doi: 10.3389/fbioe.2023.1216171 (PMC10300444; doi:10.3389/fbioe.2023.1216171)
Supplement: Supplementary file 1 [file DataSheet1.PDF]

frontiers

Supplementary Material

Table S1 The accession numbers of the obtained twelve isolates

Strain ID	Accession number
RII-2	LC769507
FU3	LC769508
FU17	LC769509
FU4	LC769510
CH3	LC769511
MY5-21	LC769512
MY3-21	LC769513
MY3-15	LC769514
MY2-9	LC769515
MY1-15	LC769516
MD4-29	LC769517
MD4-5	LC769518

All data were deposited in the DNA Data Bank of Japan.
